# HLA-E expression in diffuse glioma: relationship with clinicopathological features and patient survival

**DOI:** 10.1186/s12883-020-01640-4

**Published:** 2020-02-17

**Authors:** Zhifeng Wu, Jingshan Liang, Zheng Wang, Aimin Li, Xing Fan, Tao Jiang

**Affiliations:** 1 Beijing Neurosurgical Institute, Capital Medical University, Beijing, China; 2 Department of Neurosurgery, Lianyungang First People’s Hospital, Xuzhou Medical University, Jiangsu, China; 3 Department of Neurosurgery, Beijing Tiantan Hospital, Capital Medical University, Beijing, China

**Keywords:** Diffuse glioma, HLA-E, Clinical outcome, Prognostic biomarker

## Abstract

**Background:**

Human leukocyte antigen-E (HLA-E) has been extensively investigated in various human cancers including glioma. However, the clinical significance of HLA-E expression in glioma patients has not been elucidated. The current study aimed to investigate the association of HLA-E expression with clinicopathological features and survival in patients with diffuse glioma.

**Methods:**

A total of 261 glioma patients were enrolled, subsequently, mRNA microarray analysis was conducted to identify the relationship of HLA-E with clinicopathological features and patient survival.

**Results:**

HLA-E was significantly overexpressed in high-grade gliomas compared to low-grade gliomas (LGGs). Moreover, HLA-E expression was significantly higher in diffuse astrocytomas than oligodendrogliomas (*p* = 0.032, t-test). Kaplan-Meier analysis showed that progression-free survival (PFS) and overall survival (OS) were significantly better in LGG patients with low HLA-E expression (*p* = 0.018 for PFS and *p* = 0.020 for OS, Log-rank test). Furthermore, HLA-E expression was identified to be an independent prognostic factor by Cox analysis (*p* = 0.020 for PFS and *p* = 0.024 for OS).

**Conclusions:**

This is the first study which identified the clinical significance of HLA-E in diffuse glioma. HLA-E expression was correlated with more aggressive tumor grade and histological type and was identified as an independent prognostic biomarker in LGG patients.

## Background

According to the 2016 World Health Organization (WHO) classification [1], the diffuse gliomas include the WHO grade II/III astrocytic tumors, the grade II/III oligodendrogliomas and the grade IV glioblastomas (GBM). They are the most frequent intracranial tumors in adults, and account for over 80% of primary brain neoplasms [2]. To date, the prognosis of patients with diffuse gliomas remains disappointing despite conventional therapies (maximal tumor resection followed by radiotherapy and concomitant/adjuvant temozolomide) [3]. In the coming era of “precision medicine”, a more precise, individualized treatment strategy based on genetic, biomarker, phenotypic, or psychosocial characteristics would be more advocatable for patients with diffuse gliomas.

“Tumor-promoting inflammation” and “avoiding immune destruction” are considered as two of the hallmarks of cancer [4]. It has been widely accepted that glioma can suppress antitumor immune responses, and the underlying mechanisms are thought to be quite complex [5, 6]. A better understanding on the mechanisms of glioma-mediated immune suppression can help us to develop more selective and effective cancer treatments, i.e. immunotherapies. So far, remarkable progress has been achieved in this field and various types of immunotherapies, such as adoptive T-cell therapies and immune checkpoint therapies, have been considered as promising new modalities for glioma treatment [7–9]. However, the mechanisms behind glioma-mediated immune suppression are still far from clear.

The major histocompatibility complex (MHC) comprises a set of genes that are essential to the immune response modulation. The human MHC, i.e. human leukocyte antigen (HLA) complex, is a 4000-kb gene complex on chromosome 6 region 6p21.31. It includes over 50 recognized genes and can be separated into three subgroups: HLA-I, HLA-II and HLA-III [10]. HLA molecules are well-known for their major roles in the modulation of human immune system, and aberrant expression of HLA molecules has been indicated in multiple types of human cancers, such as breast cancer, prostate cancer and non-small cell lung cancer [11–13]. The association between HLA and glioma was investigated early in 1978, the authors determined HLA-typing of 80 glioma patients and compared the antigen frequencies with normal controls, but no significant results were found [14]. However, in recent decades, some significant findings on the relationship of HLA with glioma have been reported. For instance, Diao et al. identified a negative correlation of HLA-DR expression to patient survival in glioma patients [15].

HLA-E and HLA-G are both representative molecules of MHC class Ib (nonclassical), which differs from MHC class Ia (classical) by its nonpolymorphic and conserved nature [16]. Our group once investigated the clinical significance of HLA-G expression in glioma patients and identified that HLA-G expression was a potential biomarker for predicting aggressive entities of glioma, moreover, it could also serve as an independent predictor of poor clinical outcomes in patients with low-grade gliomas (LGG, WHO grade II) [17, 18]. Accordingly, we hypothesized that HLA-E might be also a potential biomarker for glioma patients to predict aggressive entities and poor clinical outcomes. To the best of our knowledge, there have been only one clinical-pathological correlative study which investigated the clinical significance of HLA-E expression in gliomas [19]. However, the research objectives were only limited to GBM patients, and the sample size was too small (39 cases). In the current study, we performed a large sample analysis to investigate the clinicopathological significance of HLA-E in human diffuse gliomas.

## Methods

### Patients and tissue samples

The data from 305 consecutive patients treated for diffuse glioma at the Glioma Center of Beijing Tiantan Hospital between June 2007 and September 2013 were retrospectively reviewed. After excluding 44 patients with secondary/recurrent tumors, 261 patients with histologically confirmed primary diffuse gliomas were eventually enrolled in the study. Subsequently, the pathological diagnosis of each enrolled patient was re-evaluated by an experienced neuropathologist according to the 2016 WHO classification, data of isocitrate dehydrogenase (IDH) mutation status (detected by pyrosequencing, 233 patients available) and 1p19q co-deletion status (detected by fluorescence, 236 patients available) were reviewed. Additionally, negative control brain tissue samples were collected from 5 patients with craniocerebral trauma. Clinical information (age, gender, etc.) of all patients were collected from the Chinese Glioma Genome Atlas database. The tissue samples were snap frozen and stored in liquid nitrogen immediately after resection until further processing. For reducing the influence of contamination, only samples with tumor cells over 80% would be selected for further analysis. The current study was approved by the Ethics Committee of Beijing Tiantan Hospital, and written informed consents were obtained from all patients.

### RNA extraction and whole genome mRNA expression profiling

RNA isolation and microarray analysis were performed as previously described [18]. Briefly, first, total RNA was isolated by MirVana miRNA Isolation kit (Thermo Fisher Scientific, Waltham, USA). Then a NanoDrop ND-1000 spectrophotometer (NanoDrop Technologies, Wilmington, USA) was subsequently used to perform the quantification of exacted RNA. Total RNA integrity was checked by an Agilent 2100 Bioanalyzer (Agilent, California, USA), Messenger RNA expression microarray was performed on all samples with the Agilent Whole Human Genome Array (Agilent, California, USA). Data acquisition was carried out by the Agilent G2565BA Microarray Scanner System and Agilent Feature Extraction Software (version 9.1). The normalization of probe intensities was performed using GeneSpring GX 11.0. Normalized gene expression value were log-transformed for analysis.

### Statistical analysis

All statistical analyses were performed with the R project for statistical computing (www.r-project.org) and GraphPad Prism (version 8.0.1 for Windows, GraphPad Software, San Diego, California, USA). Variables were compared using Chi-square test, Student t-test, Mann-Whitney U test, or Fisher exact test as appropriate, and a *p*-value < 0.05 was considered statistically significant. Continuous variables were expressed by mean and standard deviation (SD). For survival analysis, patients were divided into two subgroups based on their HLA-E mRNA expression values (cut off at 50% of the group). Kaplan-Meier method (Log-rank test) was performed to investigate the correlation of HLA-E mRNA expression levels with progression-free survival (PFS) and overall survival (OS) in each tumor grade. PFS was defined as the time from surgery until tumor recurrence (identified by neuroimaging according to the McDonald or the Response Assessment in Neuro-Oncology criteria), while OS was defined as the time from surgery until death or date of last follow-up. At last, Cox regression analysis was used to further evaluate the prognostic value of the identified markers.

## Results

### Clinical characteristics

Clinical information and mRNA expression microarray data of 261 patients were obtained. Among these patients, 117 were diagnosed as LGGs (62 diffuse astrocytomas, IDH-mutant, 22 diffuse astrocytomas, IDH-wildtype; 7 diffuse astrocytomas, NOS; 12 oligodendrogliomas, IDH-mutant and 1p19q-codeleted; 14 oligodendrogliomas, NOS), 36 as anaplastic gliomas (AG, WHO Grade III; 13 anaplastic astrocytomas, IDH-mutant; 14 anaplastic astrocytomas, IDH-wildtype; 3 anaplastic oligodendrogliomas, IDH-mutant and 1p19q-codeleted; 5 anaplastic oligodendrogliomas, NOS; 1 anaplastic oligoastrocytoma, NOS), 108 as GBMs (12 GBMs, IDH-mutant; 81 GBMs, IDH-wildtype; 15 GBMs, NOS). The clinical characteristics of all patients are shown in Table 1.

**Table 1:** Clinical characteristics of enrolled patients with diffuse gliomas (*N* = 261)

Characteristics	Grade II (*N* = 117)	Grade III (*N* = 36)	Grade IV (*N* = 108)
Median age (range, yrs)	38 (18–61)	42.5 (18–66)	50.5 (13–70)
Gender (male)	69	21	66
Side (Left)	58	17	58
KPS > 80	70	16	33
Pathology			
Diffuse astrocytoma/GBM, IDH mutant	62	13	12
Diffuse astrocytoma/GBM, IDH wildtype	22	14	81
Diffuse astrocytoma/GBM, NOS	7		15
Oligodendroglioma, IDH mutant and 1p19q co-deleted	12	3	
Oligodendroglioma, NOS	14	5	
Oligoastrocytoma, NOS		1	

*KPS* Karnofsky performance score*, IDH* isocitrate dehydrogenase

### HLA-E mRNA expression in diffuse gliomas

**Fig. 1:**
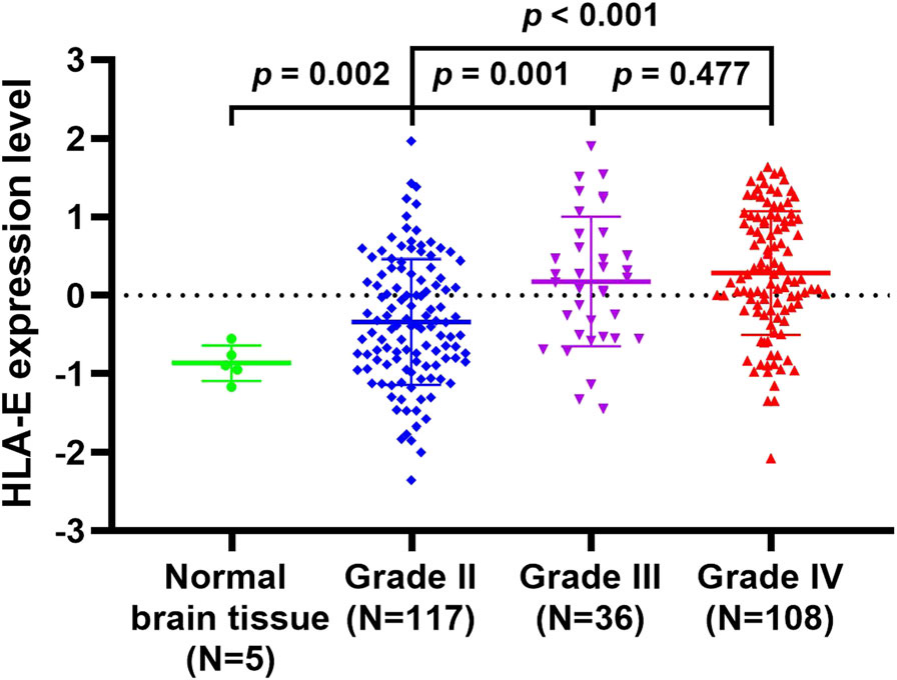
HLA-E expression levels in normal samples (*N* = 5) and different grades of gliomas (*N* = 261)

HLA-E expression in normal brain tissues and diffuse glioma tissues of each grade are shown in Fig. 1. A significant positive correlation of HLA-E expression and tumor grade was identified, HLA-E gene was highly overexpressed in GBMs (0.286 ± 0.787), followed by AGs (0.176 ± 0.824) and LGGs (− 0.340 ± 0.802). Even in LGGs, the expression of HLA-E was significantly higher than it in normal brain tissues (− 0.340 ± 0.802 versus − 0.863 ± 0.228, *p* = 0.002, t-test, Fig. 1). Significant differences in HLA-E expression could be observed between low-grade and high-grade glioma (HGG, Grade III and IV) tissues (*p* = 0.001 and *p* <  0.001, t-test, LGGs versus AGs and LGGs versus GBMs, respectively, Fig. 1), while no significant difference in HLA-E expression was identified between AGs and GBMs (*p* = 0.477, t-test, Fig. 1). Expression levels of HLA-E in 5 normal brain tissues are summarized in Supplementary Table S1.

**Fig. 2:**
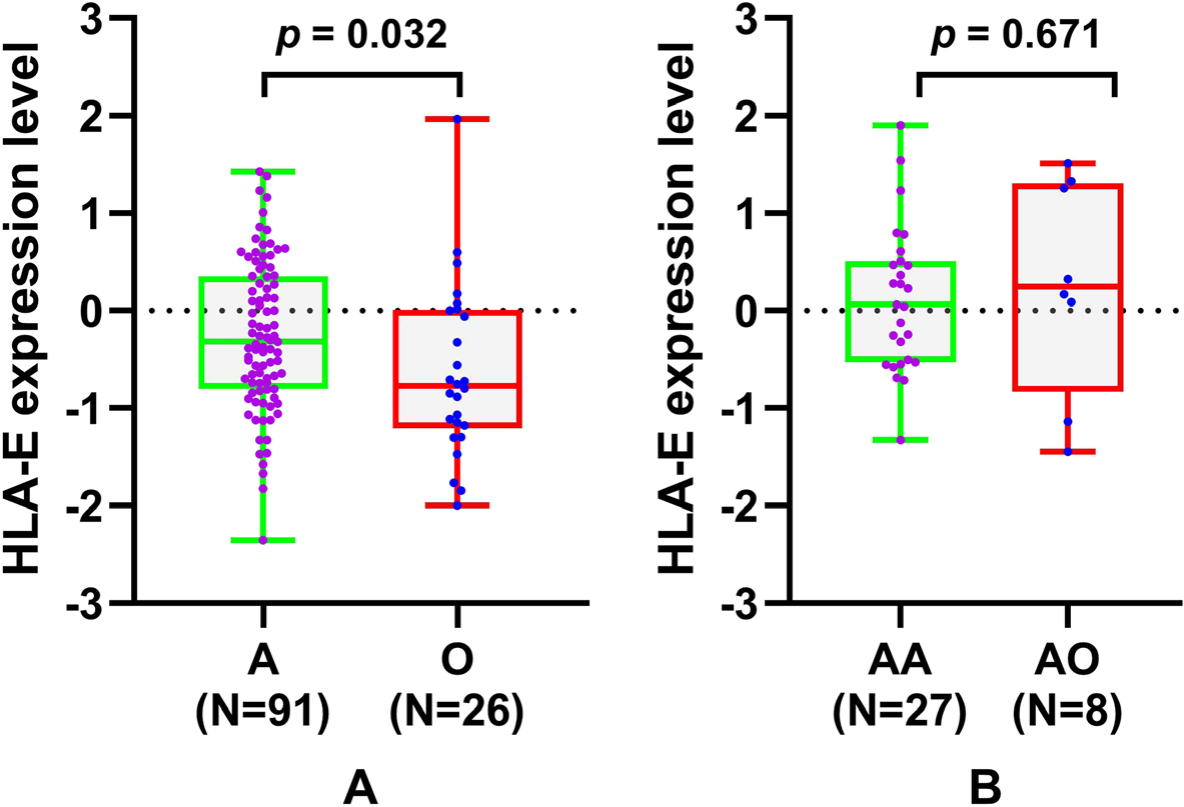
Comparison of HLA-E mRNA expression between astrocytic tumors and oligodendroglial tumors in low-grade gliomas and anaplastic gliomas A, astrocytoma; O, oligodendroglioma; AA, anaplastic astrocytoma; AO, anaplastic oligodendroglioma.

To further identify the clinical significance of HLA-E expression in diffuse glioma, we also compared HLA-E mRNA expression between astrocytic tumors and oligodendroglial tumors in LGGs and AGs. We found that HLA-E expression was significantly higher in diffuse astrocytomas than it in oligodendrogliomas (− 0.255 ± 0.762 versus − 0.636 ± 0.879, *p* = 0.032, t-test, Fig. 2a), while no such difference was observed between anaplastic astrocytomas and anaplastic oligodendrogliomas (*p* = 0.671, t-test, Fig. 2b).

Additionally, we also compared HLA-E mRNA expression levels according to IDH1 mutation status in diffuse gliomas of each grade, no association between HLA-E expression and IDH1 mutation status was identified (Grade II, *p* = 0.830; Grade III, *p* = 0.400; Grade IV, *p* = 0.412, t-test, Fig. S1).

### Survival analysis

**Fig. 3:**
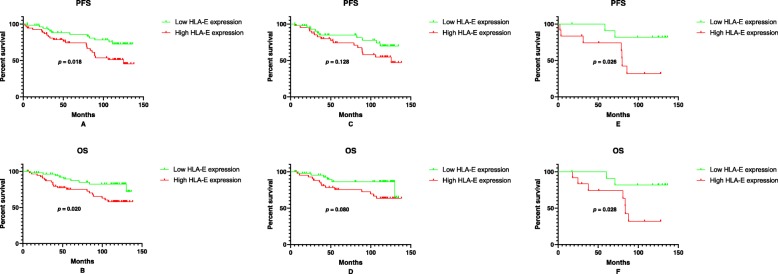
Kaplan-Meier survival analysis for patients with low-grade gliomas^a^. The figure shows comparisons of (**a**) PFS between high and low HLA-E expression group in LGG patients (*p* = 0.018); (**b**) OS between the two subgroups in LGG patients (*p* = 0.020); (**c**) PFS between the two subgroups in patients with diffuse astrocytomas (*p* = 0.128); (**d**) OS between the two subgroups patients with diffuse astrocytomas (*p* = 0.080); (**e**) PFS between the two subgroups in patients with oligodendrogliomas (*p* = 0.026); (**f**) OS between the two subgroups patients with oligodendrogliomas (*p* = 0.028). PFS, progression-free survival; OS, overall survival. ^a^Results of Log-rank test.

The follow-up rate was 96.6% with 9 patients lost follow-up. The mean follow-up time of the 252 patients was 54.9 months (range 0.9–138.1 months). Kaplan-Meier analysis was performed to investigate the prognostic value of HLA-E mRNA expression. For patients with LGGs, those with low HLA-E expression tended to have significantly longer PFS (*p* = 0.018, Log-rank test, Fig. 3a) and OS (*p* = 0.020, Log-rank test, Fig. 3b). In order to further investigate the predictive value of HLA-E expression in LGG patients, we performed Kaplan-Meier analysis in patients with diffuse astrocytomas and oligodendrogliomas respectively. For patients with diffuse astrocytomas, lower HLA-E expression still seemed to correlate with better clinical outcomes, but the results were not statistically significant (*p* = 0.128 for PFS and *p* = 0.080 for OS, Log-rank test, Fig. 3c and d). As for patients with oligodendrogliomas, low HLA-E expression remained to be associated with improved PFS (*p* = 0.026, Log-rank test, Fig. 3e) and OS (*p* = 0.028, Log-rank test, Fig. 3f). In contrast, no significant differences were observed with respect to either PFS or OS between patients with low and high HLA-E expression in patients with AGs or GBMs (Fig. S2).

### Cox regression analysis

As the previous Kaplan-Meier analyses identified that HLA-E mRNA expression served as a prognostic factor for patients with LGGs rather than patients with AGs or GBMs, Cox regression analysis was performed only in patients with LGGs, and HLA-E expression was enrolled as a continuous variable. HLA-E expression was identified as an independent predictor for poor clinical outcomes (*p* = 0.020 and 0.024, for PFS and OS, respectively, Table 2). Meanwhile, Karnofsky performance score (*p* <  0.001 both for PFS and OS, Table 2) and age at diagnosis (*p* = 0.002 and 0.005, for PFS and OS, respectively, Table 2) were also identified as independent predictive factors for the clinical outcomes of LGG patients.

**Table 2:** Predictors of progression-free survival and overall survival on multivariate analysis in patients with low-grade gliomas^a^

Parameters	PFS			OS		
	Risk ratio	95% CI	*p*-value	Risk ratio	95% CI	*p*-value
HLA-E expression	1.717	1.088–2.710	**0.020**	1.778	1.080–2.926	**0.024**
Age	1.065	1.023–1.109	**0.002**	1.072	1.022–1.125	**0.005**
Sex (male)	0.909	0.452–1.839	0.790	1.484	0.681–3.233	0.321
KPS	0.899	0.862–0.938	**< 0.001**	0.906	0.866–0.947	**< 0.001**
Side (Left)	0.899	0.452–1.788	0.761	1.317	0.608–2.853	0.486

*PFS* Progression-free survival*, OS* overall survival, *LGG* low-grade glioma*, CI* confidence interval, *KPS* Karnofsky performance score

^a^ Results of Cox regression analysis

## Discussion

Glioma is considered as a disease accompanied by profound genomic alterations. In recent decades, great progress has been made in understanding the molecular pathological basis of glioma. The identification of tumor-specific genetic alterations, such as IDH mutation, 1p19q co-deletion, telomerase reverse transcriptase promoter mutation, etc. has refreshed our understanding of this lethal disease [20, 21]. However, the understanding about the molecular information of glioma remains insufficient. For instance, the role of immune-related molecular changes in glioma has not been fully elucidated.

HLA-E is one of the most extensively investigated MHC class Ib molecules and plays a double role in both innate and adaptive immunity. Aberrant expression of HLA-E has been identified in various human malignancies, such as breast cancer, gastric cancer, rectal cancer, and colorectal cancer [22–26]. As for glioma, HLA-E has been reported to be elevated in human glioblastomas by immunohistochemistry, which was in consistent with what we demonstrated by microarray analysis in this study [19]. Additionally, previous studies also explored the role HLA-E played in the occurrence and development of glioma. It has been identified that HLA-E up-regulation in glioma cells could result in enhanced resistance to NK cell-mediated immune response [27], moreover, the interaction of HLA-E on glioma cells with CD94/NKG2A (a human NK cell inhibitory receptor on lymphocytes) could also compromise innate anti-tumor immune responses [28].

In the current study, we systematically evaluated HLA-E mRNA expression in adult diffuse gliomas and found that HLA-E mRNA expression in diffuse glioma tissues was significantly higher than that in normal brain tissues, additionally, the expression level of HLA-E increased as the tumor grade progressed. Such result suggested that glioma cells had strong ability in immune resistance, which increased with the elevation of tumor grade. Additionally, our analysis also identified that the expression pattern of HLA-E was also associated with the origin of tumor cells. Compared with oligodendrogliomas, diffuse astrocytomas showed higher expression of HLA-E. It showed that diffuse astrocytomas had stronger ability in immune resistance and therefore more aggressive than oligodendrogliomas, which was in conformity to our clinical knowledge.

The association between HLA-E expression and IDH1 mutation was also investigated. IDH1 mutation has been identified to be one of most important molecular pathological alterations in glioma, but HLA-E showed little association with it. This suggested that the HLA-E driven progression might be independent of IDH status in diffuse glioma.

The prognostic value of HLA-E has been verified in various of cancers. For instance, expression of HLA-E has been reported to resulted in a worse relapse-free period for patients with breast cancer [22]. Another study demonstrated that overexpression of HLA-E was related to a lower five-year survival rate in patients with gastric cancer [25]. Consistent with these findings in other cancers, we also identified a correlation of lower HLA-E mRNA expression with better PFS and OS in LGG patients. Furthermore, HLA-E expression was identified as an independent prognostic factor of clinical outcomes for LGG patients by Cox analysis. As for HGG, Kren et al. once reported an unexpected positive correlation of HLA-E expression to patient survival in 39 cases of GBM [19]. However, here in 36 cases of AG and 108 cases of GBM, no associations between HLA-E expression and patient survival were identified. One potential explanation is that the interaction between the immune system and tumor was relatively slow, and HGG progresses so rapidly that the immune system has no enough time to exert its influence on tumors. Moreover, a previous study showed that the cellular cytotoxicity mediated by Cetuximab, an anti-epidermal growth factor receptor monoclonal antibody, could be inhibited by HLA-E membrane expression in colon cancer cells [29]. Accordingly, immunotherapy may show better efficacy on LGG patients with low HLA-E expression, especially for those with oligodendrogliomas.

The current study had its limitations. First, all the HLA-E expression data are from microarray with no experimental verification. Moreover, data are exclusively from CGGA, which may add the systematic bias. Further studies are needed to verify the results, and experimental studies should be performed to investigate the role of HLAs in the development of glioma.

## Conclusions

In the current study, by analyzing mRNA expression microarray data of 261 patients with diffuse gliomas, the correlations of HLA-E expression with tumor grade and histological type in diffuse glioma were identified. Moreover, HLA-E expression was found to be negatively related to clinical outcomes and could serve as an independent prognostic factor in patients with LGGs. Further investigation into the roles of HLA-E and other HLA molecules in glioma can help us to obtain a better understanding of the interaction between glioma and the immune system and promote the development of relevant immunotherapy.

## Supplementary information

**Additional file 1: Table S1.** HLA-E expression levels in negative controls (5 normal brain samples). **Figure S1.** HLA-E mRNA expression levels according to IDH1 mutation status in diffuse gliomas of each grade. **Figure S2.** Kaplan-Meier survival curves**.** No correlations were identified between HLA-E expression and (A) PFS; (B) OS in patients with AGs, while no correlations were identified between HLA-E expression and (C) PFS; (D) OS in patients with GBMs^a^.

## Data Availability

The dataset analyzed during the current study is available in the Chinese Glioma Genome Atlas database (http://www.cgga.org.cn/download.jsp, Part D), and the dataset ID is “mRNA-array”.

## Abbreviations

AG: Anaplastic glioma
GBM: Glioblastoma
HLA: Human leukocyte antigen
IDH: Isocitrate dehydrogenase
LGG: Low-grade glioma
MHC: Major histocompatibility complex
OS: Overall survival
PFS: Progression-free survival
SD: Standard deviation
WHO: World Health Organization
