# Supplementary material for: HLA-E expression in diffuse glioma: relationship with clinicopathological features and patient survival
Source: BMC Neurol. 2020 Feb 17;20:59. doi: 10.1186/s12883-020-01640-4 (PMC7025409; doi:10.1186/s12883-020-01640-4)

**Table S1** HLA-E expression levels in negative controls (5 normal brain samples)

| Samples | HLA-E expression |
| --- | --- |
| 1 | -0.7744436 |
| 2 | -0.7974701 |
| 3 | -0.7208338 |
| 4 | -0.5625839 |
| 5 | -0.6984205 |

**Fig. S1** HLA-E mRNA expression levels according to IDH1 mutation status in diffuse gliomas of each grade.


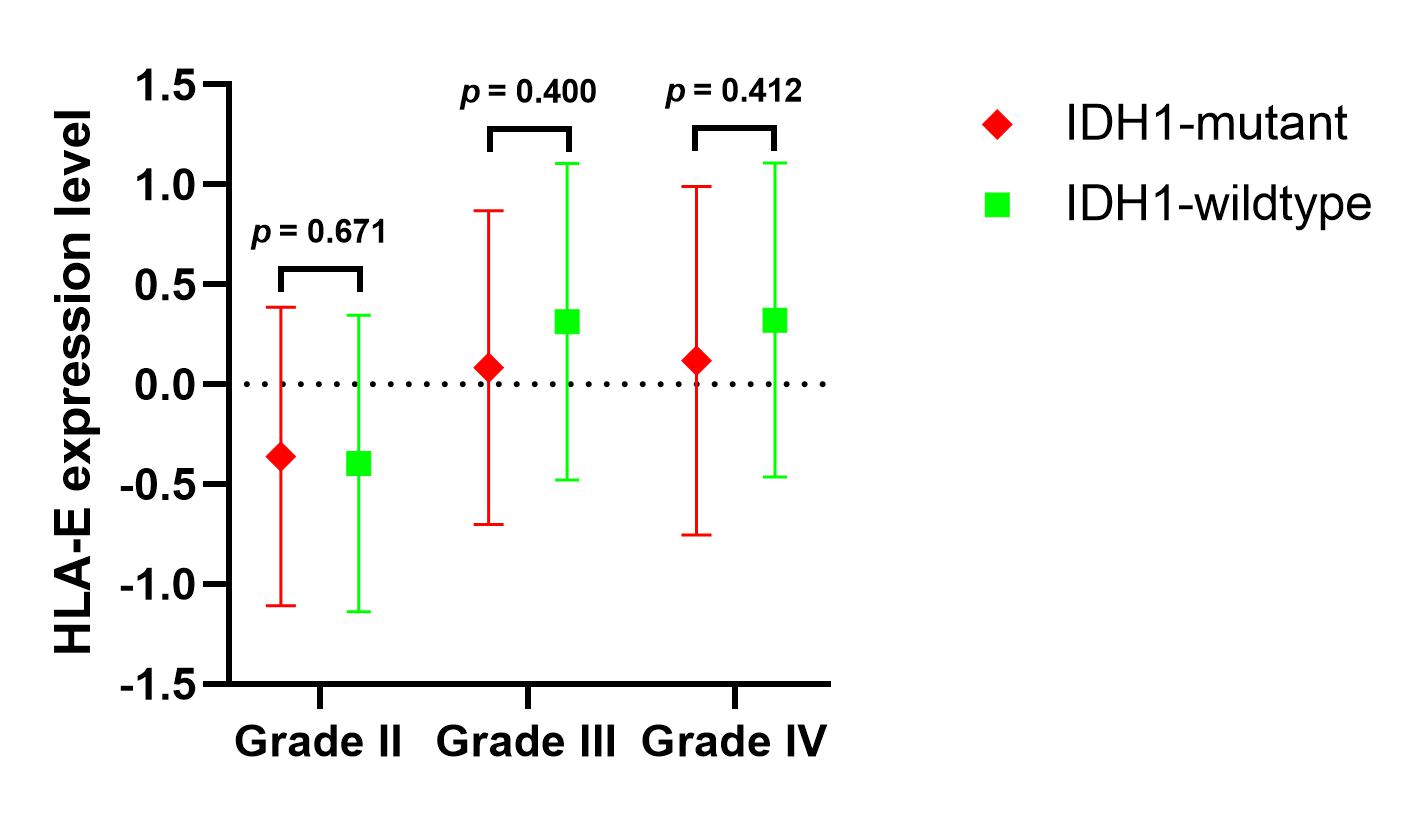


**Fig. S2** Kaplan-Meier survival curves**.** No correlations were identified between HLA-E expression and (A) PFS; (B) OS in patients with AGs, while no correlations were identified between HLA-E expression and (C) PFS; (D) OS in patients with GBMs^a^.

*PFS, progression-free survival; OS, overall survival*

^a^ Results of Log-rank test.


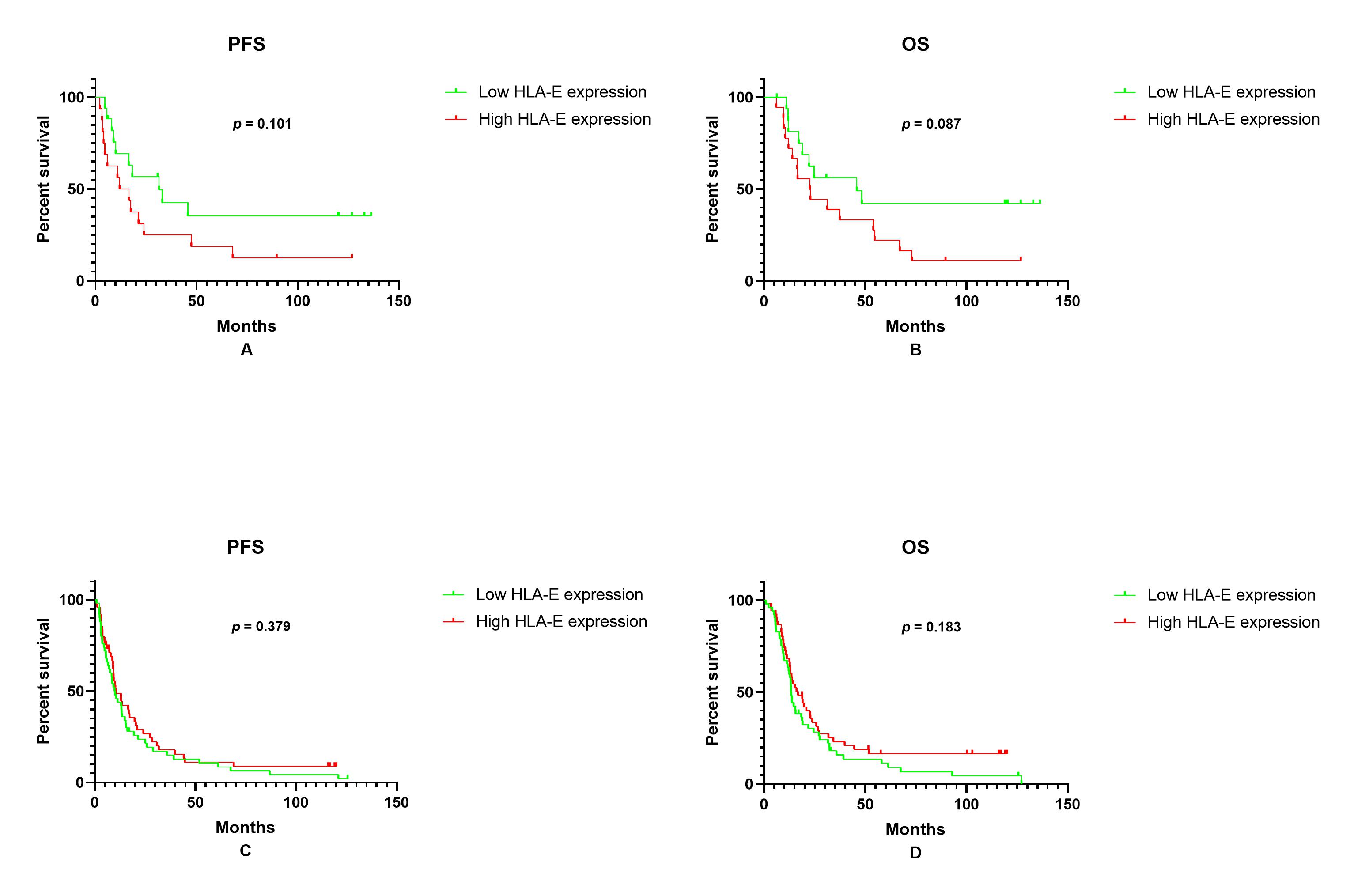

Supplement: Supplementary file 1 — Additional file 1: Table S1. HLA-E expression levels in negative controls (5 normal brain samples). Figure S1. HLA-E mRNA expression levels according to IDH1 mutation status in diffuse gliomas of each grade. Figure S2. Kaplan-Meier survival curves. No correlations were identified between HLA-E expression and (A) PFS; (B) OS in patients with AGs, while no correlations were identified between HLA-E expression and (C) PFS; (D) OS in patients with GBMsa. [file 12883_2020_1640_MOESM1_ESM.doc]
